# Prescribing Patterns of Isotretinoin for Acne Among Dermatologists in Central Jordan

**DOI:** 10.7759/cureus.58840

**Published:** 2024-04-23

**Authors:** Ruba F Al-Sheyab, Diala Alshiyab, Rawan A Al-Shagarin, Rand Murshidi, Husam A ALSalamat, Haya Abu-Rayyan, Yasmine Abu-Arja, Sumaia Ghunaim, Shawkat Altamimi

**Affiliations:** 1 Division of Dermatology, Department of Internal Medicine, Faculty of Medicine, Al-Balqa Applied University, Al-Salt, JOR; 2 Department of Dermatology, Faculty of Medicine, Jordan University of Science and Technology, Irbid, JOR; 3 Department of Dermatology, Al-Hussein New Salt Hospital, Ministry of Health, Al-Salt, JOR; 4 Department of Dermatology, School of Medicine, University of Jordan, Amman, JOR; 5 Department of Biopharmaceutics and Clinical Pharmacy, School of Pharmacy, University of Jordan, Amman, JOR; 6 Department of Special Surgery, Faculty of Medicine, Al-Balqa Applied University, Al-Salt, JOR

**Keywords:** jordan, acne, isotretinoin, prescribing pattern, dermatologists

## Abstract

Introduction: Prescribing practices among dermatologists play a crucial role in managing acne, particularly concerning medications like isotretinoin. In Jordan's central region, encompassing the Governorates of Amman, Balqa, Zarqa, and Madaba, dermatologists in both public and private sectors encounter diverse cases of acne. Understanding their prescription patterns and awareness regarding isotretinoin usage is essential for optimizing acne treatment outcomes and minimizing potential risks.

Methods: This study aimed to evaluate dermatologists' practices in prescribing isotretinoin for acne. It relied on the descriptive analytical approach, with the study population including all dermatologists working in the public and private sectors in the central region of Jordan. Simple random sampling was used to include 147 male and female doctors. An online questionnaire was adopted to collect data from the study sample, which was distributed through social media platforms and messaging platforms such as Facebook, WhatsApp, and Instagram to dermatologists working in the central region.

Results: In this study of 147 dermatologists, 58 (39.45%) prescribed isotretinoin primarily for severe acne, and 53 (36.06%) prescribed isotretinoin to about 50-100 patients per year, with the initial dosage based on guidelines (n=102, 69.39%). The majority (n=115; 78.23%) refrained from prescribing if liver enzymes were elevated. Pregnancy tests were required by 42 (28.57%) in the first session, while 78 (53.07%) deemed it the patient's responsibility. Common precautions included sunscreen (n=77; 52.38%) and moisturizing cream (n=31, 21.09%). Only six of the dermatologists (4.08%) advised their patients not to use contact lenses, and only 17 (11.57%) prescribed moisturizing eye drops.

Conclusion: This study's findings emphasize how crucial physicians' experience is when it comes to prescribing isotretinoin for severe acne. Continued educational initiatives are imperative to address gaps in patient information and safeguards in order to optimize treatment outcomes and ensure patient safety.

## Introduction

Acne vulgaris is a persistent, prevalent inflammation of the pilosebaceous unit that often arises during puberty and may or may not subside as puberty ends [1]. While males are more susceptible to severe forms in adulthood, females tend to develop acne vulgaris more frequently as they age [2]. Its global incidence is 9.38% across all age groups, and it is ranked eighth among the most common skin disorders in the world. The incidence varies between countries and age groups, with estimates ranging from 35% to nearly 100% of adolescents suffering from acne at some point. Acne is prevalent among almost all individuals aged 15 to 17 years, and a large proportion (about 15-20%) suffers from moderate to severe forms of the condition [3]. Acne commonly occurs in areas involving a large number of sebaceous glands, such as the face, proximal upper extremities, trunk, and neck [4]. The severity of the disease can range from types that heal on their own without leaving scars to severe forms that leave complications such as hyperpigmentation and mainly atrophic scars. Complications can negatively affect the quality of life and self-image, leading to higher rates of anxiety, depression, and even suicidal thoughts [5].

Acne can also be the main manifestation of many syndromes, for example, congenital adrenal hyperplasia and polycystic ovary syndrome which are related to androgens and insulin resistance, respectively. The understanding of this association will help in revealing more about the pathogenesis of acne and guiding its therapy [6].

The main goal of treatment is to improve the appearance and prevent scarring while reducing the possibility of psychological distress [4]. Topical agents are preferred for treating mild to moderate acne due to the low incidence of adverse reactions. Systemic agents may be necessary in cases where topical treatments are ineffective or when severe acne is present [2].

Although many treatments are available, isotretinoin is unique in that it is a prescription-only drug that is derived from Vitamin A, which is very effective and can lead to long-lasting improvement in acne. Oral isotretinoin was approved in 1982 by the US Food and Drug Administration for the treatment of acne vulgaris [7]. It reduces the activity and size of the sebaceous gland, normalizes the keratinization of sebaceous follicles, reduces the number of Propionibacterium acne [8], and treats various causes of acne effectively by reducing inflammation, comedones, and keratinization of the follicles and sebum production. It also reduces neutrophil chemotaxis and the amount of cutaneous acne in the follicles [9]. Isotretinoin can be associated with a variety of side effects, including photosensitivity, dry eyes, dry skin and mucosa, and bone and muscle symptoms. In particular, there have been debates and concerns about whether or not isotretinoin is related to psychiatric illness, including depression and suicide [10-12]. A systematic review and meta-analysis showed that there is no significant difference in depression rates between the users of isotretinoin and those on other treatment options [13]. Another global study focuses on the psychological impact of isotretinoin compared to oral antibiotics, and it reveals that isotretinoin has an even lower risk of depression than oral antibiotics and nearly equal rates of suicidal attempts to those who are on oral antibiotics [14].

Isotretinoin has the potential to effectively treat acne, but there are many controversial opinions about this medication that are often based on insufficient data [15]. Literature indicates that there is a low level of awareness among users about the possible side effects of isotretinoin, the duration of treatment, and some instructions about the use of isotretinoin [16]. A study showed that there are no specific criteria to determine the appropriate dose of low-dose isotretinoin [17]. To improve the use of isotretinoin, many studies and systematic reviews have suggested reducing the initial and daily doses while prolonging the duration of treatment for an additional month after acne clearance; this is not only to improve recovery rates and reduce relapses but also to reduce the prevalence of adverse events and to increase treatment adherence [18].

In light of this, this study aimed to identify the attitudes and practices of dermatologists in the central region of Jordan, regarding the prescription of isotretinoin for the treatment of acne.

## Materials and methods

Study population and sample

This study relied on the descriptive analytical approach, as the study population included dermatologists who are working in the public and private sectors in the central region of Jordan (Amman Governorate, Balqa Governorate, Zarqa Governorate, and Madaba Governorate), and the study sample is formed using a simple random sampling method to include 147 male and female doctors. The dermatologists who are currently working in the central region and who give their consent to participate in this study are included. Those who did not give their consent to participate are excluded from this study. Wherever guidelines are referred to, dermatologists in Jordan rely on two major resources for the management of acne, *Dermatology *by Bolognia et al., and the "Guidelines of Care for the Management of Acne Vulgaris" published by the American Academy of Dermatology. Both of these resources sufficiently cover all aspects of acne management using isotretinoin, including the iPLEDGE-mandated Risk Evaluation and Mitigation Strategy aimed to prevent isotretinoin exposure in pregnancy [19, 20].

Study tool

The study used the questionnaire as a study tool to collect data from the study sample, and it relied on a group of previous studies in formulating the questionnaire [21, 22], which consists of 10 dimensions and multiple answers. It was created through Gmail forms and distributed through various social media platforms such as (Facebook, WhatsApp, and Instagram) to the dermatologists who are currently working in the central region of Jordan.

To ensure the robustness of the study findings, the questionnaire was assessed for validity and reliability. Looking at the data presented in Table 1, we find that the value of the Cronbach Alpha Coefficient test ranged between (0.785-0.904), and the general index for all items was (0.936), all of which are greater than (0.70), so the study tool can be described as reliable and bear the characteristic of internal consistency.

**Table 1:** Results of reliability coefficients for the study tool dimensions according to the Cronbach Alpha test. Cronbach's alpha of >0.70 expresses acceptable internal consistency reliability.

Dimension/variable	Number of items	Cronbach Alpha
Number of patients	4	0.810
Reasons for starting treatment	6	0.805
Reasons for refusing to prescribe medication	7	0.785
Isotretinoin dosage	4	0.823
When to take pregnancy tests	4	0.790
The best ways to prevent pregnancy	6	0.817
Patients with a previous history of depression	4	0.794
Blood tests after starting treatment	7	0.803
How long is isotretinoin prescribed?	6	0.801
Precautions for side effects	6	0.904
The general indicator of reliability for all items of the study tool	54	0.936

Ethical consideration

This study was performed in line with the principles of the Declaration of Helsinki. Approval was granted by the Institutional Review Board (IRB) Committee of Al-Balqa Applied University. All participants were informed and consented to participate in the study and approved of the publication of its findings. All participants had the option to stop their participation throughout the data collection process, and they were prompted about their right to withdraw from the study anytime by communicating with the corresponding author. No personal data of study participants were obtained. Identifiable data of participants were coded and anonymized prior to formal analysis. The study data were secure and only accessible by the authors of the study through strict privileged access to ensure confidentiality and privacy.

Statistical analysis

The obtained information was statistically analyzed using SPSS version 20 (IBM Corp., Armonk, NY). The normal distribution of the data was examined using the Comaroff-Shapiro test. Categorical variables are reported as frequencies and percentages. The precise number of patients for each variable having more than 0.5% missing data is indicated in the corresponding table. P-values less than 0.05 were taken into account for statistical significance.

## Results

A total of 147 dermatologists participated in this study. According to their answers, 53 (36.06%) dermatologists prescribed isotretinoin to 50-100 patients, and severe acne was provided the most important reason for prescribing isotretinoin (n=58; 39.45%). Notably, 115 (78.23%) of the participants reported that if the blood test showed an elevation of liver enzymes at baseline or follow-up, they would not prescribe this medication to the patient. In the majority of cases (n=102; 69.39%), the dose of isotretinoin was determined according to the recommended initial dose (the range for dose observed in our study is 0.5-2 mg/kg/day), followed by the severity of acne (n=74; 50.34%), then weight (n=66; 44.89%), and finally sex (n=14; 9.52%).

Among the participants, 42 (28.57%) indicated that the patient was asked to perform pregnancy tests in the first session only, while 78 (53.07%) participants believed that it was the patient's responsibility, and they didn’t ask them to perform the test. Intrauterine devices (IUDs) were among the most commonly prescribed methods of contraception (n=49; 33.35%). It was noted that 96 (65.31%) participants would not prescribe the medication if there was a possibility of depression (by getting information from the patient in accordance with the DSM-5 criteria for depression and with the assistance of a psychologist). Moreover, 43 (29.26%) participants requested blood tests (complete blood count, liver function test, and fasting lipid panel) every three months after starting the treatment (patients who are fertile must utilize two approved methods of contraception or maintain full abstinence for at least one month prior to, during, and following isotretinoin treatment). The interval that is required to repeat the treatment course was from one to three months according to most participants (n=108; 73.46%), and the most important precautions of the use of this drug were the use of sunscreen (n=77; 52.38%), followed by moisturizing skin cream (n=31; 21.09%), then moisturizing eye drops (n=17; 11.57%), then analgesics for joints and muscles (n=10; 6.80%), and nasal moisturizer (n=6; 4.08%), and not using contact lenses (n=6; 4.08%). Table 2 shows dermatologists' responses regarding the starting of isotretinoin to treat acne.

**Table 2:** Dermatologists’ responses regarding starting isotretinoin for acne treatment (n=147). N, frequency number; %, percentage

The dimension	Item	N(%)
The number of patients who had an isotretinoin prescription in the last year (2022)	0–10	20 (13.60)
	10–50	33 (22.45)
	50–100	53 (36.06)
	More than 100	41 (27.89)
Important reasons for starting treatment with isotretinoin	Treating patients with skin disorders	5 (03.40)
	Failure to respond to previous treatments	22 (14.97)
	An indicator of resistant nodular acne	45 (30.61)
	According to my experience, I prescribe it to people who suffer from severe acne	58 (39.45)
	Patient request	5 (03.40)
	Psychological complications resulting from acne	12 (08.16)
Causes of refusal to prescribe isotretinoin	A blood test indicates liver enzyme abnormality	115 (78.23)
	Unreliable contraceptives	45 (03.61)
	Central nervous system disorders	15 (10.20)
	Current depression	7 (04.80)
	Previous history of depression	7 (04.80)
	Lipid profile abnormality	39 (26.53)
	Pregnancy and fertility problems	72 (48.99)
The dose of isotretinoin is determined according to:	The weight	66 (44.89)
	General initial dose	102 (69.39)
	Severity of acne	74 (50.34)
	Sex	14 (09.52)
When do you ask the patient to perform pregnancy tests?	Every month before the prescription	12 (08.16)
	First session only	42 (28.57)
	With periods of more than a month	15 (10.20)
	Never, it is the patient's responsibility	78 (53.07)
What is the most effective method of contraception?	Barrier contraceptives	17 (11.57)
	Oral contraceptive drugs (OCP)	33 (22.45)
	Intrauterine devices (IUD)	49 (33.35)
	Long-term contraceptives such as progesterone injections	29 (19.73)
	I will not prescribe anything	8 (05.44)
	Depends on the patient	11 (07.48)
Do you refer patients with a prior history of depression to a specialist before starting isotretinoin?	No	22 (14.97)
	Yes	9 (06.12)
	If there is a possibility of depression, I will not prescribe isotretinoin	96 (65.31)
	Depends on the patient	20 (13.60)
When do you request blood tests after starting treatment?	Per month	6 (04.08)
	Once after 6-8 weeks	12 (08.16)
	Every three months	43 (29.26)
	Every two months	34 (23.13)
	Periods exceeding three months	41 (27.89)
	After a month and then every two months	5 (03.40)
	None of the above	6 (04.08)
Repeated prescribing of isotretinoin course	Less than a month	12 (08.16)
	From one to three months	108 (73.46)
	Three to six months	12 (08.16)
	Six to nine months	7 (04.80)
	Nine to twelve months	5 (03.40)
	More than twelve months	3 (02.04)
Precautions for side effects	Moisturizing eye drops	17 (11.57)
	Not using contact lenses	6 (04.08)
	Use sunscreen	77 (52.38)
	Use moisturizing skin cream	31 (21.09)
	Nasal moisturizer	6 (04.08)
	Analgesic for joints and muscles	10 (06.8)

## Discussion

Patients with acne vulgaris should be treated primarily by dermatologists and this research aims to shed light on prescribing patterns of one of the most used modalities of treatment for acne, which is isotretinoin. The decision to start using isotretinoin emphasizes the need for a dermatologist's examination, and the necessity to perform tests before starting treatment, especially a comprehensive blood test, liver function, and blood lipids, and this is extremely important to avoid exacerbation of the side effects of this drug [22]. The use of isotretinoin for an average duration of 50 days or more, results in elevated levels of triglycerides, cholesterol, and liver enzymes [23]. The elevations in lipids and liver enzymes return to baseline values shortly after cessation of treatment [24].

The success of using the medication depends on the experience of the treating physician and their understanding of the patient’s condition, circumstances, quality of life, and the ability of the treating physician to follow up on the patient’s health and psychological condition, thus ensuring a safe and effective treatment process. The predictors of acne recurrence after the end of treatment include younger age, prior history of acne before adolescence, a family history of moderate to severe acne, widespread acne in the body, and inadequate dosing in the preceding isotretinoin course [25].

In the Jordanian context, it seems necessary for dermatologists to sharpen their understanding of isotretinoin, including its potential side effects and associated conditions, by raising the level of awareness of potential side effects, and the urgent need to take the necessary precautions for side effects. In this study, only a few of the participating dermatologists advised the patients not to use contact lenses (n=6, 4.08%), and few prescribed moisturizing eye drops (n=17; 11.57%); in any case, the most important side effect of the drug is teratogenicity [26].

It is necessary to refer patients to specialists in case the initial assessment revealed an established history of depression or suspicion of depression before and during isotretinoin treatment [27], but our results indicated that 22 (14.97%) of participants didn’t refer patients with a previous history of depression to a specialist before starting isotretinoin, and 20 (13.60%) relied on the patient’s decision to do so; this may be explained by the negative view and social culture prevailing in Jordanian society about the psychologists, and the unwillingness of individuals to go to them. 

It is necessary to focus more on the most effective method of contraception, which is thought to be hormonal with either combined oral contraceptive pills or hormonal injections or implantation that should be considered one month before isotretinoin initiation, during the full duration of treatment and continuing for at least one month after cessation of treatment [28]. All women of childbearing age should be aware of the teratogenicity of this drug [28]. In our study, 78 (53.07%) of participants thought that doing a pregnancy test was the patient's responsibility. This is not always reliable because not all patients are well-educated or know enough about isotretinoin's impact on pregnancy and it is the healthcare provider's responsibility to educate patients about this drug and its side effects. It’s worth mentioning that there are restrictions on its use due to the risk of teratogenicity in many countries, including New Zealand, the United Kingdom, and Australia [29].

Limitations of this study include concerns about recall bias and generalizability of the study findings, as the participants might not represent the general population of dermatologists in Jordan. Therefore, we recommend performing multicenter studies to obtain broader insights into the practice of isotretinoin initiation to treat acne among dermatologists. Additionally, some precautions regarding the side effects of the use of isotretinoin might not have been addressed like hair loss, blood transfusion, suicidal ideation, and over-strenuous activity. Also, the study focused mainly on a one-year timeframe; an extended prospective timeframe would provide a better understanding of prescribing patterns.

## Conclusions

This study concludes that in the central region of Jordan, dermatologists' education is crucial when prescribing isotretinoin for severe acne. Despite the sizable portion of doctors prescribing isotretinoin, there are noticeable gaps in patient information and preventative measures, such as recommending against the use of contact lenses and prescription moisturizing eye drops. It is imperative to address contraindications such as increased liver enzymes and possible depression. Additionally, dermatologists should comply with guidelines and educate patients about alarming symptoms associated with isotretinoin. In order to maximize treatment outcomes and patient safety, going forward, dermatologists must have a better awareness of the use of isotretinoin, including its side effects and related precautions. This will require further education initiatives within the dermatological community.
